# 1-Chloro-1-[(4-meth­oxy­phen­yl)hydrazinyl­idene]propan-2-one

**DOI:** 10.1107/S1600536811026389

**Published:** 2011-07-09

**Authors:** Abdullah M. Asiri, Abdulrahman O. Al-Youbi, Mohie E. M. Zayed, Seik Weng Ng

**Affiliations:** a Chemistry Department, Faculty of Science, King Abdulaziz University, PO Box 80203 Jeddah, Saudi Arabia; b The Center of Excellence for Advanced Materials Research, King Abdul Aziz University, PO Box 8020 Jeddah, Saudi Arabia; c Department of Chemistry, University of Malaya, 50603 Kuala Lumpur, Malaysia

## Abstract

The non-H atoms of the title compound, C_10_H_11_ClN_2_O_2_, lie nearly on a plane (r.m.s. deviation = 0.150 Å), and the C=N double bond has a *Z* configuration. In the crystal, adjacent mol­ecules are linked by an N—H⋯O_carbon­yl_ hydrogen bond, forming a chain running along [201].

## Related literature

For the synthesis, see: Benincori *et al.* (1990 ▶); Sayed *et al.* (2002 ▶). For background to the title compound, see: Asiri *et al.* (2010 ▶).
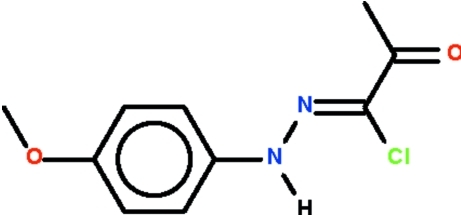


## Experimental

### 

#### Crystal data

C_10_H_11_ClN_2_O_2_
*M* *_r_* = 226.66
Monoclinic, 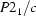

*a* = 5.8873 (3) Å
*b* = 25.0467 (10) Å
*c* = 7.3041 (3) Å
β = 99.016 (4)°
*V* = 1063.74 (8) Å^3^
*Z* = 4
Cu *K*α radiation
μ = 3.05 mm^−1^
*T* = 100 K
0.35 × 0.10 × 0.05 mm

#### Data collection

Agilent SuperNova Dual diffractometer with an Atlas detector
Absorption correction: multi-scan (*CrysAlis PRO*; Agilent, 2010 ▶) *T*_min_ = 0.415, *T*_max_ = 0.863
3802 measured reflections
2090 independent reflections
1776 reflections with *I* > 2σ(*I*)
*R*_int_ = 0.026

#### Refinement

*R*[*F* ^2^ > 2σ(*F* ^2^)] = 0.038
*wR*(*F* ^2^) = 0.102
*S* = 1.05
2090 reflections
142 parameters
H atoms treated by a mixture of independent and constrained refinement
Δρ_max_ = 0.25 e Å^−3^
Δρ_min_ = −0.30 e Å^−3^

### 

Data collection: *CrysAlis PRO* (Agilent, 2010 ▶); cell refinement: *CrysAlis PRO*; data reduction: *CrysAlis PRO*; program(s) used to solve structure: *SHELXS97* (Sheldrick, 2008 ▶); program(s) used to refine structure: *SHELXL97* (Sheldrick, 2008 ▶); molecular graphics: *X-SEED* (Barbour, 2001 ▶); software used to prepare material for publication: *publCIF* (Westrip, 2010 ▶).

## Supplementary Material

Crystal structure: contains datablock(s) global, I. DOI: 10.1107/S1600536811026389/xu5259sup1.cif

Structure factors: contains datablock(s) I. DOI: 10.1107/S1600536811026389/xu5259Isup2.hkl

Supplementary material file. DOI: 10.1107/S1600536811026389/xu5259Isup3.cml

Additional supplementary materials:  crystallographic information; 3D view; checkCIF report

## Figures and Tables

**Table 1:** Hydrogen-bond geometry (Å, °)

*D*—H⋯*A*	*D*—H	H⋯*A*	*D*⋯*A*	*D*—H⋯*A*
N2—H2⋯O1^i^	0.87 (3)	2.22 (3)	3.021 (2)	153 (2)

Symmetry code: (i) 
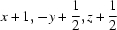
.

**Fig. 1.**
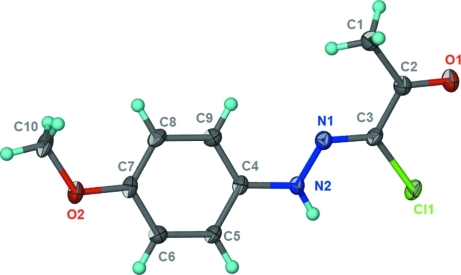
Thermal ellipsoid plot (Barbour, 2001) of C10H11N2O2 at the 70% probability level; hydrogen atoms are drawn as spheres of arbitrary radius.

## supplementary crystallographic information

### Comment

We have previously reported the synthesis of ethyl (*Z*)-2-chloro-2-(2-phenylhydrazin-1-ylidene) acetate by the reaction of benzenediazonium chloride with ethyl 2-chloro-3-oxobutanoate (Asiri *et al.*, 2010). The compound is an ester. In the present study, the use of a substituted benzenediazonium chloride and the methyl ester (instead of the ethyl ester) afforded a 1-chloro-1-(arylhydrazono)-2-propanone. Such ketones are intermediates in the synthesis of pyrazoles (Sayed *et al.*, 2002) and other heterocycles (Benincori *et al.*, 1990). In the 4-methoxy substituted compound (Scheme I, Fig. 1), the non-hydrogen atoms lie on a plane [r.m.s. deviation 0.150 Å] (Scheme I, Fig. 1). The C_aryl_–N(H)–N═ C(S)═O portion adopts an extended zigzag conformation. Adjacent molecules are linked by an *N*–*H*···O_carbonyl_ hydrogen bond to form a chain running [2 0 1].

### Experimental

To a stirred solution of methyl 2-chloro-3-oxobutanoate (1.64 g, 10 mmol) in ethanol (100 ml) was added sodium acetate trihydrate (1.30 g, 10 mmol). The mixture was chilled to 273 K and then treated with a cold solution of *p*-nitrobenzenediazonium chloride, prepared by diazotizing *p*-methoxyaniline (1.23 g, 10 mmol) dissolved in 6*M* hydrochloric acid (6 ml) with a solution of sodium nitrite (0.70 g, 10 mmol) in water (10 ml). The addition of the diazonium salt solution was carried out with rapid stirring over a period of 20 min. The reaction mixture was stirred for further 15 min. and left for 3 h in refrigerator. The resulting solid was collected by filtration and washed thoroughly with water. The crude product was crystallized from ethanol to give the corresponding hydrazonoyl chloride.

### Refinement

Carbon-bound H-atoms were placed in calculated positions [C—H 0.95 to 0.98 Å, *U*_iso_(H) 1.2 to 1.5*U*_eq_(C)] and were included in the refinement in the riding model approximation.

The amino H-atom was located in a difference Fourier map, and was freely refined.

### Crystal data

**Table:**

C_10_H_11_ClN_2_O_2_	*F*(000) = 472
*M**_r_* = 226.66	*D*_x_ = 1.415 Mg m^−^^3^
Monoclinic, *P*2_1_/*c*	Cu *K*α radiation, λ = 1.54184 Å
Hall symbol: -P 2ybc	Cell parameters from 1678 reflections
*a* = 5.8873 (3) Å	θ = 3.5–74.0°
*b* = 25.0467 (10) Å	µ = 3.05 mm^−^^1^
*c* = 7.3041 (3) Å	*T* = 100 K
β = 99.016 (4)°	Prism, yellow
*V* = 1063.74 (8) Å^3^	0.35 × 0.10 × 0.05 mm
*Z* = 4	

### Data collection

**Table:**

Agilent SuperNova Dual diffractometer with an Atlas detector	2090 independent reflections
Radiation source: SuperNova (Cu) X-ray Source	1776 reflections with *I* > 2σ(*I*)
Mirror	*R*_int_ = 0.026
Detector resolution: 10.4041 pixels mm^-1^	θ_max_ = 74.2°, θ_min_ = 3.5°
ω scan	*h* = −5→7
Absorption correction: multi-scan (*CrysAlis PRO*; Agilent, 2010)	*k* = −20→31
*T*_min_ = 0.415, *T*_max_ = 0.863	*l* = −8→7
3802 measured reflections	

### Refinement

**Table:**

Refinement on *F*^2^	Primary atom site location: structure-invariant direct methods
Least-squares matrix: full	Secondary atom site location: difference Fourier map
*R*[*F*^2^ > 2σ(*F*^2^)] = 0.038	Hydrogen site location: inferred from neighbouring sites
*wR*(*F*^2^) = 0.102	H atoms treated by a mixture of independent and constrained refinement
*S* = 1.05	*w* = 1/[σ^2^(*F*_o_^2^) + (0.0508*P*)^2^ + 0.2595*P*] where *P* = (*F*_o_^2^ + 2*F*_c_^2^)/3
2090 reflections	(Δ/σ)_max_ = 0.001
142 parameters	Δρ_max_ = 0.25 e Å^−^^3^
0 restraints	Δρ_min_ = −0.30 e Å^−^^3^

### Fractional atomic coordinates and isotropic or equivalent isotropic displacement parameters (Å^2^)

**Table:**

	*x*	*y*	*z*	*U*_iso_*/*U*_eq_	
Cl1	0.79949 (8)	0.222023 (16)	0.49001 (6)	0.02053 (15)	
O1	0.3432 (2)	0.23571 (5)	0.2668 (2)	0.0248 (3)	
O2	1.2583 (2)	0.53251 (5)	0.8487 (2)	0.0247 (3)	
N1	0.7372 (3)	0.32750 (6)	0.4928 (2)	0.0162 (3)	
N2	0.9408 (3)	0.33231 (6)	0.5974 (2)	0.0165 (3)	
H2	1.023 (4)	0.3046 (10)	0.634 (4)	0.038 (7)*	
C1	0.3119 (3)	0.33074 (8)	0.2551 (3)	0.0240 (4)	
H1A	0.3295	0.3380	0.1263	0.036*	
H1B	0.3820	0.3597	0.3348	0.036*	
H1C	0.1481	0.3282	0.2646	0.036*	
C2	0.4288 (3)	0.27892 (7)	0.3161 (3)	0.0184 (4)	
C3	0.6546 (3)	0.28185 (7)	0.4355 (3)	0.0168 (4)	
C4	1.0117 (3)	0.38372 (7)	0.6642 (2)	0.0155 (4)	
C5	1.2413 (3)	0.39119 (7)	0.7442 (3)	0.0182 (4)	
H5	1.3455	0.3620	0.7566	0.022*	
C6	1.3153 (3)	0.44152 (7)	0.8052 (3)	0.0202 (4)	
H6	1.4710	0.4467	0.8599	0.024*	
C7	1.1642 (3)	0.48447 (7)	0.7872 (3)	0.0179 (4)	
C8	0.9350 (3)	0.47698 (7)	0.7091 (3)	0.0185 (4)	
H8	0.8307	0.5062	0.6972	0.022*	
C9	0.8601 (3)	0.42636 (7)	0.6485 (3)	0.0186 (4)	
H9	0.7037	0.4210	0.5960	0.022*	
C10	1.1081 (4)	0.57745 (7)	0.8382 (3)	0.0242 (4)	
H10A	1.1951	0.6090	0.8875	0.036*	
H10B	0.9851	0.5704	0.9112	0.036*	
H10C	1.0408	0.5838	0.7086	0.036*	

### Atomic displacement parameters (Å^2^)

**Table:**

	*U*^11^	*U*^22^	*U*^33^	*U*^12^	*U*^13^	*U*^23^
Cl1	0.0247 (3)	0.0108 (2)	0.0250 (3)	0.00228 (17)	0.00048 (18)	0.00008 (17)
O1	0.0238 (7)	0.0152 (7)	0.0340 (8)	−0.0046 (6)	−0.0002 (6)	−0.0032 (6)
O2	0.0255 (7)	0.0124 (6)	0.0348 (8)	−0.0011 (6)	0.0000 (6)	−0.0050 (6)
N1	0.0174 (7)	0.0134 (7)	0.0174 (8)	−0.0007 (6)	0.0016 (6)	0.0004 (6)
N2	0.0169 (8)	0.0101 (7)	0.0211 (8)	−0.0006 (6)	−0.0010 (6)	−0.0004 (6)
C1	0.0216 (10)	0.0160 (9)	0.0321 (11)	0.0011 (8)	−0.0032 (8)	−0.0006 (8)
C2	0.0185 (9)	0.0147 (9)	0.0214 (9)	−0.0011 (7)	0.0018 (7)	−0.0002 (7)
C3	0.0179 (9)	0.0107 (9)	0.0220 (9)	0.0013 (7)	0.0035 (7)	0.0007 (7)
C4	0.0190 (9)	0.0113 (8)	0.0162 (9)	−0.0007 (7)	0.0033 (7)	−0.0003 (7)
C5	0.0181 (9)	0.0120 (9)	0.0241 (10)	0.0018 (7)	0.0020 (7)	−0.0006 (7)
C6	0.0167 (9)	0.0172 (9)	0.0260 (10)	0.0009 (7)	0.0014 (7)	−0.0022 (8)
C7	0.0241 (10)	0.0116 (9)	0.0180 (9)	−0.0014 (7)	0.0035 (7)	−0.0014 (7)
C8	0.0207 (9)	0.0127 (9)	0.0221 (9)	0.0044 (7)	0.0029 (7)	0.0014 (7)
C9	0.0172 (9)	0.0169 (9)	0.0213 (9)	0.0018 (7)	0.0019 (7)	−0.0006 (8)
C10	0.0355 (11)	0.0102 (9)	0.0268 (11)	0.0010 (8)	0.0047 (9)	−0.0005 (8)

### Geometric parameters (Å, °)

**Table:**

Cl1—C3	1.7395 (18)	C4—C9	1.385 (2)
O1—C2	1.224 (2)	C4—C5	1.398 (3)
O2—C7	1.371 (2)	C5—C6	1.384 (3)
O2—C10	1.426 (2)	C5—H5	0.9500
N1—C3	1.287 (2)	C6—C7	1.389 (3)
N1—N2	1.322 (2)	C6—H6	0.9500
N2—C4	1.417 (2)	C7—C8	1.393 (3)
N2—H2	0.87 (3)	C8—C9	1.392 (3)
C1—C2	1.503 (2)	C8—H8	0.9500
C1—H1A	0.9800	C9—H9	0.9500
C1—H1B	0.9800	C10—H10A	0.9800
C1—H1C	0.9800	C10—H10B	0.9800
C2—C3	1.473 (3)	C10—H10C	0.9800
			
C7—O2—C10	117.36 (15)	C6—C5—H5	120.3
C3—N1—N2	122.09 (16)	C4—C5—H5	120.3
N1—N2—C4	118.27 (15)	C5—C6—C7	120.67 (18)
N1—N2—H2	121.2 (18)	C5—C6—H6	119.7
C4—N2—H2	120.3 (18)	C7—C6—H6	119.7
C2—C1—H1A	109.5	O2—C7—C6	115.38 (17)
C2—C1—H1B	109.5	O2—C7—C8	124.65 (17)
H1A—C1—H1B	109.5	C6—C7—C8	119.96 (17)
C2—C1—H1C	109.5	C9—C8—C7	119.43 (17)
H1A—C1—H1C	109.5	C9—C8—H8	120.3
H1B—C1—H1C	109.5	C7—C8—H8	120.3
O1—C2—C3	120.66 (17)	C4—C9—C8	120.53 (18)
O1—C2—C1	121.88 (18)	C4—C9—H9	119.7
C3—C2—C1	117.45 (16)	C8—C9—H9	119.7
N1—C3—C2	119.81 (16)	O2—C10—H10A	109.5
N1—C3—Cl1	122.97 (15)	O2—C10—H10B	109.5
C2—C3—Cl1	117.21 (13)	H10A—C10—H10B	109.5
C9—C4—C5	119.98 (17)	O2—C10—H10C	109.5
C9—C4—N2	121.38 (17)	H10A—C10—H10C	109.5
C5—C4—N2	118.63 (16)	H10B—C10—H10C	109.5
C6—C5—C4	119.42 (17)		
			
C3—N1—N2—C4	−175.92 (16)	C4—C5—C6—C7	−0.2 (3)
N2—N1—C3—C2	−178.79 (16)	C10—O2—C7—C6	−178.14 (16)
N2—N1—C3—Cl1	0.3 (3)	C10—O2—C7—C8	2.5 (3)
O1—C2—C3—N1	−176.92 (18)	C5—C6—C7—O2	−178.62 (17)
C1—C2—C3—N1	4.3 (3)	C5—C6—C7—C8	0.8 (3)
O1—C2—C3—Cl1	3.9 (3)	O2—C7—C8—C9	178.87 (17)
C1—C2—C3—Cl1	−174.89 (14)	C6—C7—C8—C9	−0.5 (3)
N1—N2—C4—C9	10.9 (3)	C5—C4—C9—C8	1.1 (3)
N1—N2—C4—C5	−167.94 (16)	N2—C4—C9—C8	−177.73 (17)
C9—C4—C5—C6	−0.7 (3)	C7—C8—C9—C4	−0.5 (3)
N2—C4—C5—C6	178.08 (17)		

### Hydrogen-bond geometry (Å, °)

**Table:**

*D*—H···*A*	*D*—H	H···*A*	*D*···*A*	*D*—H···*A*
N2—H2···O1^i^	0.87 (3)	2.22 (3)	3.021 (2)	153 (2)

Symmetry codes: (i) *x*+1, −*y*+1/2, *z*+1/2.
